# Predictive factors for referral to a peer support worker in psychosocial rehabilitation centers

**DOI:** 10.3389/fpsyt.2025.1648718

**Published:** 2025-10-13

**Authors:** Romain Monnier, Julien Plasse, Frédéric Haesebaert, Benjamin Gouache, Emilie Legros-Lafarge, Nathalie Guillard-Bouhet, Marie-Cécile Bralet, Nicolas Franck, Dora Khattech, Guillaume Barbalat, Céline Giraudet

**Affiliations:** ^1^ Université Claude Bernard Lyon 1 – Domaine de Rockefeller, Lyon, Rhône, France; ^2^ Centre Hospitalier Spécialisé Le Vinatier, Pôle Centre Rive Gauche, Centre Ressource de Réhabilitation Psychosociale (CRR), Bron, Rhône, France; ^3^ Centre Hospitalier Alpes-Isère, Centre Référent de Réhabilitation Psychosociale et de Remédiation Cognitive (C3R), Saint-Egrève, Isère, France; ^4^ Centre Hospitalier Esquirol, Centre Référent de Réhabilitation Psychosciale de Limoges (C2RL), Haute-Vienne, Limoges, France; ^5^ Centre Hospitalo-Universitaire de Poitiers, Centre de Réhabilitation d'Activités Thérapeutiques Intersectoriel de la Vienne (CREATIV), Poitiers, Vienne, France; ^6^ Centre Hospitalier Isarien de Clermont de l'Oise, Centre Support de Remédiation Cognitive et de Réhabilitation Psychosociale - Sud des Hauts de France (CRISALID), Clermont, Oise, France

**Keywords:** psychosocial rehabilitation, recovery-oriented practices, recovery, peer support (PS), peer support workers, predictive factors, referral

## Abstract

**Introduction:**

Peer support workers are individuals with personal lived experience of mental health conditions, addictions, or neurodevelopmental disorders, and can be employed as professionals within mental health services. This study aims to identify predictive factors for patient referral to peer support intervention in psychosocial rehabilitation services.

**Methods:**

Using data from the French REHABase cohort, we compared variables between patients referred (n=134) and not referred (n=242) to peer support intervention. We evaluated an expert-based model (clinician-selected variables) against a machine-based model (algorithm-selected variables) for predictive accuracy.

**Results:**

The machine-based model outperformed the expert-based model in the full dataset (AUC = 0.78 vs 0.71). However, the predictive performance of both models substantially declined after cross-validation, yielding modest AUC values (0.60 and 0.59), which constitutes a key limitation of the study.

**Discussion:**

Neurodevelopmental disorder diagnosis, social isolation, and low treatment adherence predicted peer support referral. Poor model performance may be due to unmeasured factors like patient motivation or clinicians’ perceptions of peer support workers.

## 1 Introduction

Peer support (PS) workers are increasingly recognized as professionals in the mental health field. They are individuals with personal lived experience of mental health conditions, addiction or neurodevelopmental disorder. They experienced crisis and recovery, and they developed strategies to cope with their situation and potential persisting symptoms. They engage with patients by drawing on this experiential knowledge. The experiential dimension, defined by Corrigan as “peerness”, distinguishes their contribution from that of other mental health professionals (1–3).

The concept of peer support in recovery emerged in the mid-19th century in the United States within small groups of former drinkers, known as “recovery circles,” who supported each other in maintaining abstinence and rebuilding meaningful lives. Various recovery groups appeared, some spiritual, others secular, or tailored to women and ethnic minorities. In 1935, Alcoholics Anonymous formalized the movement, which rapidly spread worldwide.

Peer support workers can work in various social, medical-social, or healthcare settings, with diverse outpatient or inpatient locations. There is a significant expansion of peer support workers within psychosocial rehabilitation services. Psychosocial rehabilitation is a mental health discipline that supports individuals with mental health conditions in achieving a satisfactory quality of life in their chosen environment. It fosters empowerment, the process by which individuals gain greater control over decisions and actions affecting their lives, in personal recovery (4).

Both, psychosocial rehabilitation services and peer support workers, are rooted in the recovery movement. Prior to the 1970s, mental health care was dominated by a biomedical model that viewed mental illness as chronic and incurable. Patients were often institutionalized for long-periods, with little recognition of their strengths or potential for change. Service users had no role in decision-making, and their lived experience was not valued in clinical practice or research. Emerging in the 1970s, the recovery movement is a service-user–led paradigm that emphasizes hopes, agency and self-determination, framing recovery as living well, with or without ongoing symptoms, rather than pursuing symptom elimination alone. In Anglo-Saxon countries, the recovery movement was driven by mental health service user advocacy, notably through figures such as Patricia Deegan and Bill Anthony (5).

The origins of peer support are also rooted in the psychiatric survivors’ movement of the 1980s. Figures such as Judi Chamberlin advocated for mutual aid, and self-organized alternatives to conventional psychiatry. This political struggle emphasized reclaiming power “by and for” service users, challenging stereotypes of dependency and chronicity (6). They promoted a paradigm shift toward empowerment. Progressively, numerous recovery-oriented services practicing psychosocial rehabilitation have emerged. Several public health policies supported this emergence (7).

The initial peer specialist certification programs began to appear in the United States in 2001 and, by 2015, psychosocial rehabilitation services employed over 30,000-peer support workers (8). In Europe, the recognition of peer support is heterogeneous. In Belgium, the “Expert du vécu” (“Expert by Experience”) service of the “SPP-Intégration-Sociale” has been operating since 2004 with European funding and employs 34 “Experts du Vécu” in 2024 (9). In the United Kingdom, peer support workers were first employed in mental health services in 2009, initially in small numbers within a few NHS Trusts. By 2019, this figure had grown to 862 peer support workers, the majority directly employed by the NHS (10). In France, the “World Health Organization collaborating center for research and training in mental health” introduced the “peer support workers program” in 2012 at Paris 8 university, which was the first French peer support training program to formally recognize the status of peer support workers (11). Since then, an increasing number of French universities have integrated peer support training programs (Lyon 1, Bordeaux, etc.). In France, training is increasingly formalized through university-based programs, including one-year modules offering blended theoretical and experiential learning. Recruitment often proceeds via structured pathways, where candidates with lived experience are recruited to a position and receive training during their first year of service. As of 2020, France counted 180 peer support workers employed in mental health services (12). Comparable initiatives are also expanding in Denmark (13), the Netherlands (14), Switzerland, and Germany (15).

Peer support workers’ interventions have demonstrated effectiveness in supporting individuals with mental health conditions. They have notably shown a positive impact on patients’ overall recovery (16), empowerment (17), and an increase in perceived hope (18). Integrating peer support workers into a patient’s care pathway improves their social functioning similarly to other standard clinical practices (19). The involvement of a peer support worker during treatment is a factor in preventing relapses after emergency department visits (20). In closed units, it serves as a protective factor against physical and chemical restraint (21). It also leads to a reduction in depressive symptoms comparable to Cognitive Behavioral Therapy according to Pfeiffer and al.’s meta-analysis regrouping various populations (22).

However, the role of peer support workers in psychiatric care remains unclear and poorly defined for some professionals (23). There are no established guidelines to assist clinicians in the process of referring a patient to peer support follow-up. The French National Authority for Health stated in a 2023 policy document that it is “necessary to define an intervention framework and a status for peer support at a national level” (24). This framework should clarify the modalities of intervention (intensity of follow-up, grouped or individual modality), the scope of action of peer support workers and the criteria for referral, including patient profiles and indications. Our study specifically addresses this latter aspect. To our knowledge, no prior publications have examined the factors associated with referral to peer support interventions. While the effectiveness of such interventions has been increasingly documented, the mechanisms underlying this effectiveness and the reasons guiding clinical referrals remain poorly understood and insufficiently explored in the international literature. A prerequisite would be to conduct an inventory of patient profiles and indications that have led to peer support follow-up. We conducted a retrospective observational study using prospectively collected data from a national cohort. The main objective of this study is to identify predictive factors for referral to peer support interventions in French psychosocial rehabilitation services. This is the first empirical inventory of patient profiles currently referred to peer support within psychosocial rehabilitation services, using a large national cohort (REHABase). It addresses a gap in the literature that has focused mainly on effectiveness rather than referral mechanisms. Identifying predictors enables the operationalization of implicit clinical heuristics into explicit referral guidance for routine triage in psychosocial rehabilitation. The results of this study could offer an evidence base for drafting fair and transparent referral criteria and for informing service policy.

## 2 Materials and methods

### 2.1 Data

#### 2.1.1 Description of database

We conducted our study using data from the French cohort REHABase. REHABase is a database containing evaluation indicators collected in psychosocial rehabilitation services. It was established in 2016 by the «Centre Ressource en Réhabilitation Psychosociale» (CRR) in Lyon. Thirty psychosocial rehabilitation centers have contributed to its data collection as of 2023. The analyzed data was from the initial assessment, in the beginning of psychosocial rehabilitation care. Participants were prospectively recruited for this study beginning in 2016, with recruitment ongoing at the time of writing. The most recent data point included in the analysis was collected on August 3, 2023. Data were gathered at multiple time points—before, during, and after care—by a multidisciplinary team including physicians, nurses, psychologists, occupational therapists, and peer support workers. Analyzed data were extracted in September 2023 and pertain to individuals receiving care at one of thirty partnering psychosocial rehabilitation centers. All participants provided verbal non-opposition, which was systematically recorded in our secure database in accordance with ethical guidelines.

Initial user care in psychosocial rehabilitation services begins with an initial assessment interview with a clinician and the completion of an initial functional and psychometric evaluation. At intake, a clinician (a psychiatrist) records clinical and psychometric data and obtains patient consent for participation in the REHABase study. If consent is given, the data are subsequently entered into the REHABase platform by the clinician or by clinical research associates. Then, the patient will be directed toward rehabilitation interventions, based on their needs and the difficulties identified during the initial assessment. These interventions may include, but are not limited to, individual or group-based cognitive remediation, psychoeducation, social skills training, supported employment, and peer support interventions.

We included participants whose initial data was collected between 2016 and 2023 and who had at least one follow up intervention documented in the cohort. During this period, 6,026 participants were included in the REHABase cohort. Among them, 1,997 participants had the initial intake and at least one following intervention documented. We excluded participants without documented intervention after baseline, those who changed centers during their course of care, and those with incomplete data – defined as having more than 45% missing data. In the control group, we also excluded participants included in centers that did not offer peer support during their follow-up and those whose primary diagnosis was different from patients in the peer support group. We excluded participants with eating disorder, somatoform disorder, sleeping disorders, elimination disorder, sexual disorder and neurocognitive disorder in order to make the two study samples more comparable.

#### 2.1.2 Outcome variable

Our outcome variable was referral to a peer support specialist. We compared two groups of patients. The first group consisted of patients who were referred to peer support intervention (PS group). The other group included participants who were not referred for peer support (nPS group; control). To form the nPS group, we matched it with the PS group based on the following criteria: 1) the care center, 2) the rank of peer support intervention among the proposed psychosocial rehabilitation interventions, and 3) the duration of participation in psychosocial rehabilitation until the peer support intervention (PS group) or until the end of follow-up (nPS group). An nPS group patient who matches a PS group patient according to these criteria will be referred to as their “neighbor.” The matching was performed at a ratio of 2 in the nPS group to 1 in the PS group. We needed a control group larger than the test group to reflect reality. Indeed, the rehabilitation center clinicians surveyed estimated that they referred one patient to peer support for every four patients encountered (resulting in a 3:1 ratio). However, our sample and our relatively restrictive selections criteria prevented us from obtaining a ratio higher than 2:1.

**Figure 1:**
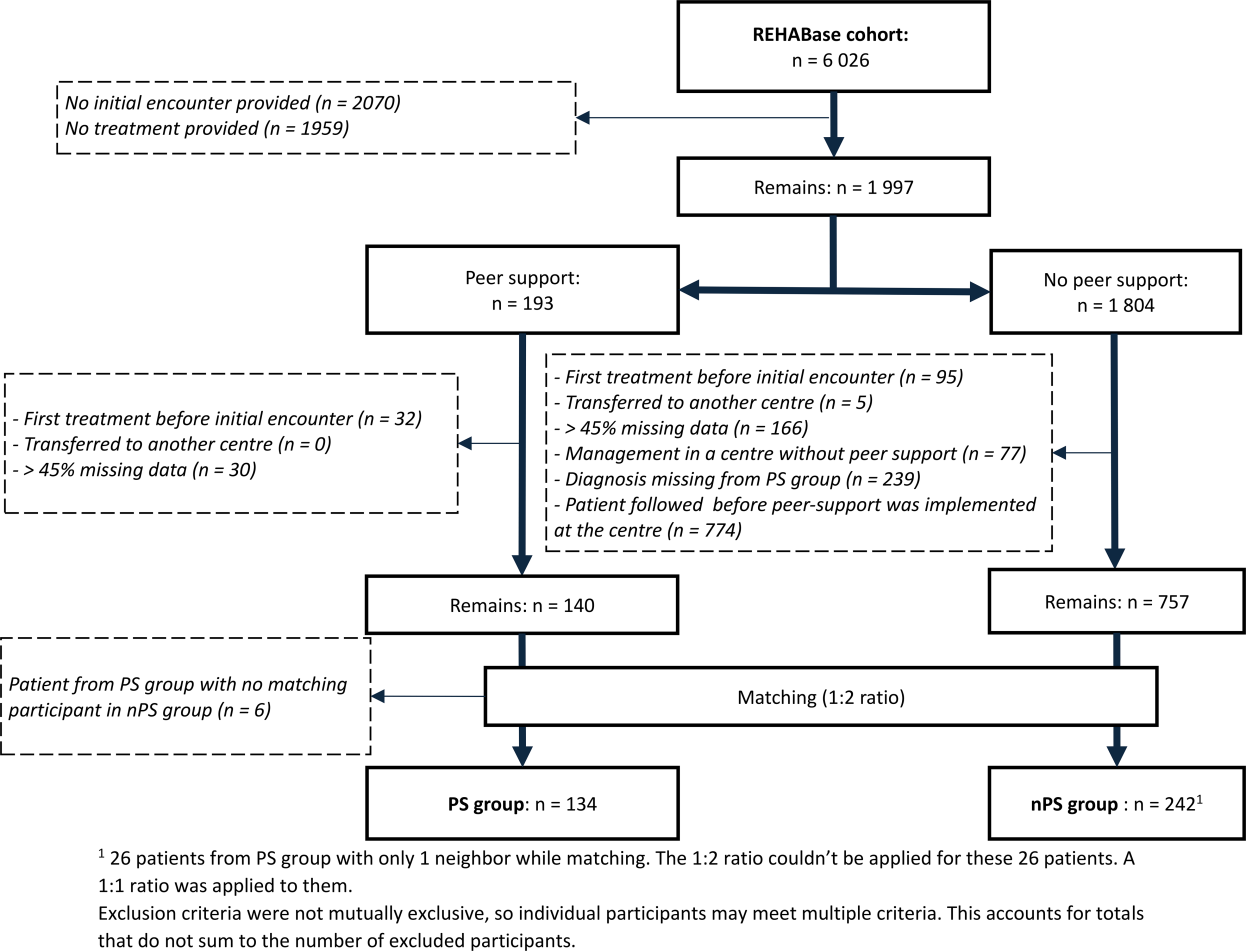
Patient selection and matching process in Peer-Supported (PS) and Non-Peer-Supported (nPS) follow-up groups. ^1 ^26 patients from PS group with only 1 neighbor while matching. The 1:2 ratio couldn’t be applied for these 26 patients. A 1:1 ratio was applied to them. Exclusion criteria were not mutually exclusive, so individual participants may meet multiple criteria. This accounts for totals that do not sum to the number of excluded participants.

After applying the exclusion criteria, the PS group included 140 patients. During the matching process, we identified six patients in the PS group whose characteristics did not match any patients in the nPS group. These six patients were excluded from the analyses. Additionally, during the matching, only one neighbor was found instead of two in the nPS group for 26 patients from the PS group. Therefore, after matching, the sample sizes for analysis, were 134 patients in the PS group and 242 patients in the nPS group. The participant selection and matching process is presented in **Figure 1**.

#### 2.1.3 Predictors

We defined variables of interest to extract from REHABase for each participant in the sample. The extracted variables include socio-demographic variables (age, sex, level of education, family situation, employment status, housing type, presence of Protection of Vulnerable Adults, presence of disability recognition, marginalization, forensic history), clinical variables (primary diagnosis, duration of illness, presence of psychiatric comorbidities, number of psychotropic medications, duration since first contact with psychiatry, number and average duration of hospitalizations, history of suicidal behaviors, presence of addictive comorbidities: tobacco, alcohol and psychoactive substances) and psychometric variables (total score at the Schizophrenia Quality of Life scale (SQoL18) (25, 26), the Global Assessment of Functioning (GAF) (27), the Clinical Global Impressions (CGI) (28), the Warwick-Edinburgh Mental Well-being Scale (WEMWBS) (29) and the Medication Adherence Rating Scale (MARS) (30), total scores and dimensions of the “Social Autonomy Scale” (EAS) (31), Self-Esteem Rating Scale (SERS) (32), Birchwood Insight Scale (BIS) (33), Internalized Stigma of Mental Illness scale (ISMI) (34), and Stages of Recovery Instrument (STORI) (35)).

### 2.2 Analysis

We conducted the analysis using the open-source statistical and graphical software R (36). Initially, we examined the differences between each variable in the PS group and the nPS group through bivariate analysis. Then, we performed a multivariate analysis using logistic regression to identify predictive factors of referral to a peer support intervention. For logistic regression, we compared two models. In the first model, the studied variables were preselected by clinicians (expert-based model), whereas in the second model, we took all available variables and let the algorithm select the most relevant ones based on the Akaike Information Criterion (AIC) (machine-based model).

We developed the expert-based model by convening a group of four clinicians to engage in discussions and debates on the most relevant criteria for directing patients to peer support workers, based on their professional experience. Alongside these expert-chosen variables, commonly used demographic factors (age, gender, marital status, employment status, etc.) frequently found in similar studies (37) were included. The final selection of variables was agreed upon through consensus among the clinicians. The variables preselected were age, sex, marital status, employment status, primary diagnosis, duration of illness, number of psychiatric hospitalizations, number of psychotropic medications, presence of personal housing, education level, history of suicidal behaviors, stage of recovery in the STORI, and total scores on the CGI, SERS, ISMI, SQol18, EGF, and MARS scales. The number of retained variables is eighteen.

The second model was constructed by minimizing the AIC as a compromise between model accuracy (goodness of fit) and interpretability (number of parameters). In other words, the AIC method helps to choose a model that explains the data well without being overly complex. It works by adding variables one by one to a base model (with only basic predictors) and calculating AIC at each step. If adding the variable lowers the AIC, it improves the model. If the AIC increases, it means that adding the new variable worsens the model.

We addressed missing data in both bivariate and multivariate analyses using imputation. This process incorporated pre-selected variables as well as auxiliary variables from the database that were not initially chosen for the primary analysis (e.g. sub scores of the ISMI). The auxiliary variables were included to improve the imputation model’s performance and reduce potential bias.

Finally, we calculated the predictive accuracy in non-cross-validated and cross-validated models. To achieve this, and considering the small sample size, the validation method used was 10-fold cross-validation (38). Cross-validation is a machine learning methodology that evaluates the performance of our model by testing it on data separate from those used during training to limit the risk of bias during testing. The data are divided into 10 subsets. The model is trained on 9 subset and tested on the remaining subset, repeating 10 times while varying the training and test sets. The average performance over these iterations was then calculated to estimate the overall model performance (39). The metric used is the Area Under the Receiver Operating Characteristic Curve (AU-ROC), with “1 - specificity” on the x-axis and sensitivity on the y-axis. For both models, we reported odds ratios and their 95% confidence intervals (CI) for each of the included variables.

### 2.3 Ethics statement

The study was authorized by the French legislation (French National Advisory Committee for the Treatment of Information in Health Research, 16.060bis), including information processing (French National Computing and Freedom Committee, DR-2017-268) (40). We obtained verbal non-opposition from the participants, and this information was duly recorded in our database.

## 3 Results

**Figure 2:**
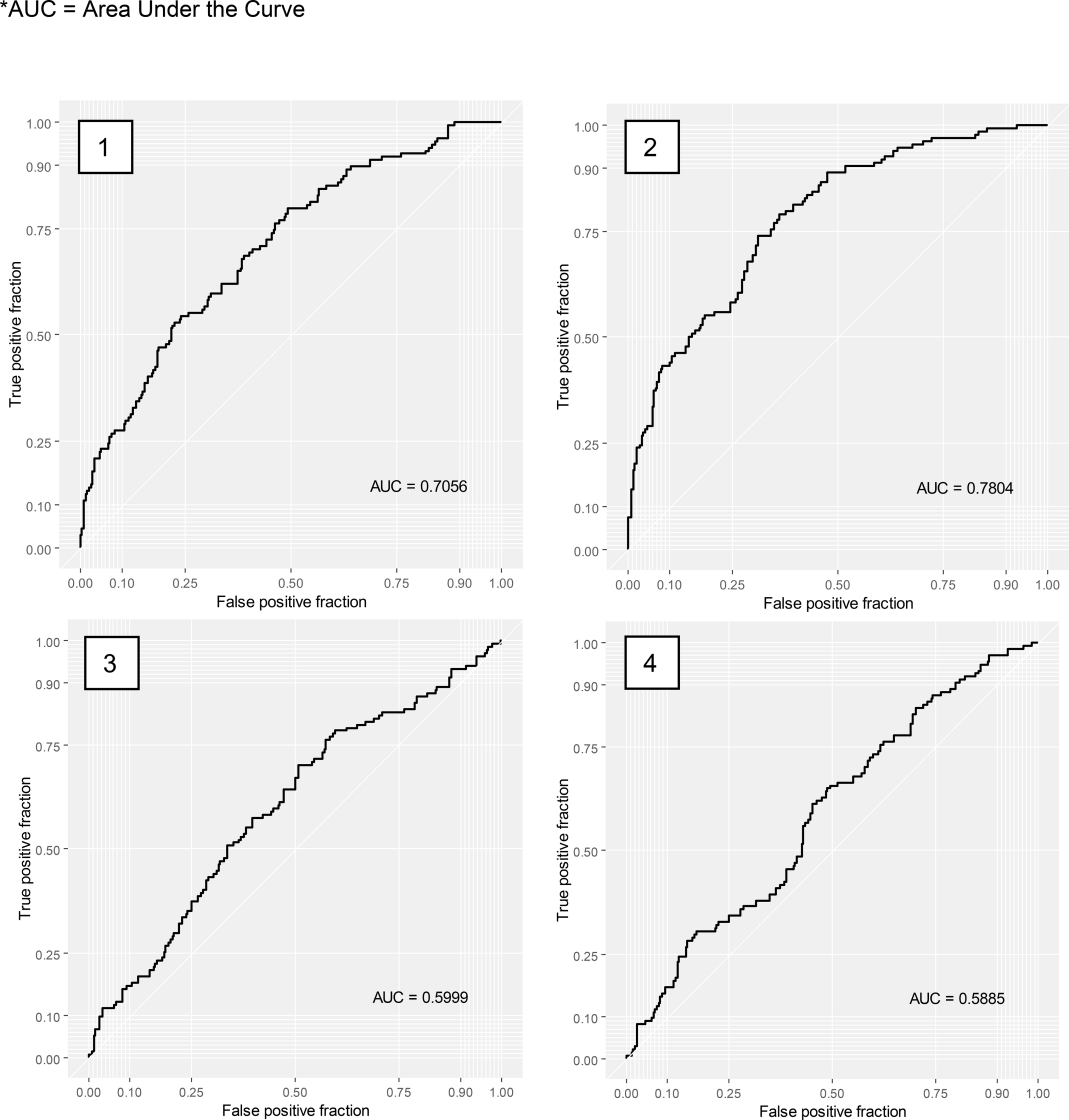
Receiver Operating Characteristic (ROC) Curve, predictive accuracy of the expert-based model (1 and 3) and the machine-based model (2 and 4) before cross-validation (1 and 2) and after cross-validation (3 and 4). *AUC, Area Under the Curve.

The characteristics of patients referred to peer support, obtained after imputation, are displayed in **Supplementary Table**. Results of the multivariate regression are shown in **Table 1** for the expert-based model and in **Table 2** for the machine-based model. The predictive accuracy of our model is illustrated in **Figure 2**.

**Table 1:** Comparison between patients oriented toward a peer support worker (PS) and not oriented toward a peer support worker (nPS), expert-based model.

Variable	OR (95% CI)^1^	p-value
Age	1,02 (0.99 to 1.05)	0.25
Female	1,15 (0.69 to 1.90)	0.59
Bachelor’s degree or higher	1.33 (0.78 to 2.29)	0.30
Primary diagnosis (DSM-5), n (%)		
*Neurodevelopmental disorder*	—	
*Schizophrenia* sp*ectrum*	0.49 (0.22 to 1.11)	0.087
*Bipolar disorder*	0.44 (0.17 to 1.13)	0.090
*Other: anxiety, depressive or personality disorder*	0.14 (0.06 to 0.33)	<0.001*
Number of treatments, n (%)		
*None*	—	
*1 or 2*	0.78 (0.38 to 1.63)	0.51
*3 or more*	0.62 (0.27 to 1.39)	0.24
GAF^2^, mean (SD)	1.03 (1.00 to 1.06)	0.025*
CGI^3^, mean (SD)	1.37 (0.98 to 1.94)	0.066
Marital status		
*Single*	—	
*In a relationship*	0.93 (0.51 to 1.68)	0.82
Duration of the disease, year	0.98 (0.95 to 1.02)	0.32
Professional situation, n (%)		
*Unemployed*	—	
*Employed*	0.91 (0.42 to 1.91)	0.81
Housing status, n (%)		
*Homeless/Hospital/Squats*	—	
*Personal home*	0.91 (0.06 to 26.0)	0.95
*Family home*	1.11 (0.07 to 32.2)	0.94
*Social care home/Others*	0.31 (0.02 to 10.1)	0.45
Number of hospitalizations, n (%)		
*None*	—	
*1-3*	1.04 (0.53 to 2.06)	0.91
*4-9*	1.79 (0.80 to 4.05)	0.16
*10 or more*	0.87 (0.16 to 3.84)	0.86
SQoL18^4^ – Total score	0.99 (0.97 to 1.00)	0.15
STORI^5^ - Moratorium	0.99 (0.96 to 1.03)	0.64
SERS^6^ – Total score	1.00 (0.99 to 1.02)	0.71
ISMI^7^ – Total score	1.50 (0.71 to 3.20)	0.29
MARS^8^ – Total score	1.04 (0.92 to 1.18)	0.53
BIS^9^ – Total score	0.94 (0.85 to 1.03)	0.18

^1^OR, Odds Ratio, CI, Confidence Interval; ^2^GAF, Global assessment of functioning; ^3^CGI, Clinical global impression; ^4^SQoL18, Schizophrenia Quality of life; ^5^STORI, Stage of recovery instrument; ^6^SERS, Self-esteem rating scale; ^7^ISMI, Internalized stigma mental illness; ^8^MARS, Medication adherence rating scale; ^9^BIS, Birchwood insight scale. Bold values indicate statistically significant results at p < 0.05.

**Table 2:** Comparison between patients oriented toward a peer support worker (PS) and not oriented toward a peer support worker (nPS), machine-based model.

Variable	OR (95% CI)^1^	p-value
Primary diagnosis (DSM-5), n (%)		
*Neurodevelopmental disorder*	—	
*Schizophrenia* sp*ectrum*	0.56 (0.31 to 1.00)	0.052
*Other: anxiety, depressive or personality disorder*	0.15 (0.07 to 0.31)	<0.001*
Secondary diagnosis, n (%)		
*No*	—	
*Yes*	1.55 (0.87 to 2.80)	0.14
GAF, mean (SD)	1.04 (1.01 to 1.07)	0.019*
CGI, mean (SD)	1.39 (0.97 to 2.01)	0.078
Housing status, n (%)		
*Homeless/Hospital/Squats*	—	
*Personal home*	3.88 (1.31 to 13.7)	0.021*
*Family home*	2.75 (0.91 to 9.77)	0.091
Recognition of disabled worker status (RQTH)		
*No*	—	
*Yes*	0.87 (0.55 to 1.37)	0.533
*Pending request*	3.52 (0.76 to 16.6)	0.10
Protection of Vulnerable Adults		
*No*	—	
*Yes*	0.28 (0.08 to 0.83)	0.029*
History of suicidal behaviors, n (%)		
*No*	—	
*Yes*	0.64 (0.35 to 1.14)	0.13
Addiction psychoactive substance, n (%)		
*No*	—	
*Yes*	0.51 (0.23 to 1.08)	0.089
SQoL18 – Level of resilience	0.99 (0.98 to 1.00)	0.11
SQoL18 – Level of physical well-being	1.02 (1.00 to 1.03)	0.009*
SQoL18 – Quality of family relationships	1.00 (0.99 to 1.01)	0.956
SQoL18 – Quality of sentimental life	0.99 (0.98 to 1.00)	0.12
SQoL18 – Level of psychological well-being	0.99 (0.98 to 1.00)	0.091
WEMWBS – Total score (z-score)	1.56 (1.17 to 2.09)	0.003*
EAS - Daily life management	1.06 (1.01 to 1.12)	0.021*
EAS - Management of external relations	0.91 (0.84 to 0.98)	0.017*
EAS - Management of emotional life and social relationships	1.10 (1.02 to 1.19)	0.015*
BIS – Need for treatment	0.76 (0.57 to 0.99)	0.045*
ISMI – Social withdrawal	2.04 (1.30 to 3.25)	0.002*
STORI – Preparation	0.97 (0.94 to 1.00)	0.030*

^1^OR, Odds Ratio, CI, Confidence Interval; GAF, Global assessment of functioning; CGI, Clinical global impression; RQTH, Reconnaissance de la qualité de travailleur handicapé; SQoL18, Schizophrenia Quality of life; WEMWBS, Warwick-Edinburg mental well-being scale; EAS, Echelle d’autonomie sociale; BIS, Birchwood insight scale; ISMI, Internalized stigma mental illness; STORI, Stage of recovery instrument. Bold values indicate statistically significant results at p < 0.05.

In the non-partitioned dataset, the expert-based model had a predictive accuracy materialized by an Area Under the Curve (AUC) of 0.71 and the machine-based model had an AUC of 0.78. Cross-validated models’ AUC were of 0.60 for the expert-based model, and 0.59 for the machine-based model. An AUC of 0.71 indicates modest discrimination: it means the model ranks a randomly chosen referred patient above a non-referred patient 71% of the time. Following common conventions, AUC values are interpreted as: 0.5 (chance level), 0.6–0.7 (low–modest), 0.7–0.8 (acceptable), 0.8–0.9 (good).

In the expert-based model (**Table 1**), the statistically significant results were as follows: patients diagnosed with Neurodevelopmental Disorder (NDDs) were 86% more likely to be referred to a peer support worker than those with anxiety-depressive disorder or personality disorder; for every 1-point increased on the Global Assessment of Functioning scale, the likelihood of being referred to a peer support worker increased by 3%.

In the machine-based model (**Table 2**), results were statistically significant for following **socio-demographic variables**: patients with personal housing had a 3.88 times higher chance of being referred to a peer support worker; patients without legal Protection of Vulnerable Adults had a 72% higher chance of being referred to a peer support worker.

In the machine based model, they were also a significant difference for these **clinical variables**: patients with a diagnosis of neurodevelopmental disorder had an 85% higher chance of being referred to a peer support worker than patients with a diagnosis of anxiety-depressive disorder or personality disorder.

Finally, in the machine based-model, results were significant for following **psychometric variables**. For each 1-point increased in the social withdrawal subscale of the Internalized Stigma of Mental Illness (ISMI), the chance of being referred to a peer support worker was multiplied by 2.04. For each 1-point increased in the total mental well-being score on the Warwick-Edinburgh Mental Well-being Scale (WEMWBS), the chance of being referred to a peer support worker increased by 56%. For each 1-point increased in the “awareness of the need for treatment” dimension on the Birchwood Insight Scale, the chance of being referred to a peer support worker increased by 24%. For each 1-point increased in the “management of external relations” dimension measured on the EAS, the chance of being referred to a peer support worker decreased by 9%. For each 1-point increased in the “difficulty in managing daily life” and “difficulty in managing emotional life and relationships” dimensions on the Social Autonomy Scale (EAS), the chance of being referred to a peer support worker increased by 6% and 10% respectively. For each 1-point increased on the Global Assessment of Functioning (GAF), the chance of being referred to a peer support worker increased by 4%. Patients in the “preparation” stage of the STORI had a 3% lower chance of being referred to a peer support worker. For each 1-point increased in level of physical well-being on the SQoL18 scale, the chance of being referred to a peer support worker increased by 2%.

## 4 Discussion

### 4.1 Data discussion

The predictive accuracy of multivariate models was acceptable before cross-validation, with an AUC of 0.71 and 0.78 for the expert-based and machine-base models respectively. However, the cross-validation step is necessary to mitigate the issue of model overfitting. An overfitting model fails to recognize new data and cannot generalize its results. After cross-validation, we decreased this overfitting issue but lost predictive accuracy, which decreased to 0.60 and 0.59. Such a low predictive accuracy suggests that other important variables were omitted. Several hypotheses can be suggested regarding the nature of these variables. On the patient side, it can be assumed that parameters such as motivation or expressed treatment request during the interview influence their allocation to a peer support program. Other extrinsic factors may be presumed, such as the diagnosis of the employed peer support worker, their training, the type of intervention offered. The availability of peer support workers at specific centers and their training or specializations can play a role. The clinicians’ perception of peer support worker profession and their level of knowledge about this profession was also omitted. Clinicians may be guided by existing research that suggests peer support is particularly beneficial for certain groups of patients, such as patients with a poor social functioning (19) or patients with depressive symptoms (22). Some clinicians may have a stronger belief in the effectiveness of peer support based on their personal experience, or they may have biases in favor of or against referring certain types of patients. To enhance the predictive accuracy of the model, further investigation could explore allocation to peer support versus a more specific intervention (e.g., cognitive behavioral therapy, cognitive remediation, or psychoeducation).

This study finds that patients diagnosed with a neurodevelopmental disorder (NDDs) are 1.85 times more likely to be referred to a peer support intervention. There is limited data on the specific impact of peer support intervention in NDDs (41). Our results do not allow for discrimination among different NDDs (autism spectrum disorder, attention deficit hyperactivity disorder, language disorder, etc.) Clinicians may perceive NDDs as particularly amenable to peer support intervention. Peer support represents an interesting no-pharmacological intervention for this population, particularly through experiential sharing and destigmatization. Conversely, patients diagnosed with anxiety-depressive disorders or personality disorders were less frequently referred to a peer support worker. It should be noted that during the study period, the Grenoble center (C2RL) employed a peer support worker with a diagnosis of autism spectrum disorder. It is highly probable that the knowledge of the colleague’s diagnosis directly or indirectly influences the clinician’s referral choice. Moreover, the Grenoble center is recognized as an expert center for NDDs with a broad regional appeal, which is not the case for the other centers. A center effect cannot be ruled out in interpreting these results. Nonetheless, this raises the question of the relevance of matching the patient’s psychiatric diagnosis with that of peer support worker, as each disorder has its own specificities. Some peer-support workers reports that they are more comfortable with certain disorders. Conversely, some patients may find it more helpful to meet a peer support worker with the same diagnosis. This could facilitate identification and enhance the perceived relevance of experiential sharing. This “diagnostic matching” may, in certain cases, strengthen the peer relationship and make recovery narratives more accessible. In everyday clinical practice, the diagnosis of peer support worker is not always the same as that of the patient, yet this does not hinder their intervention. In fact, the underlying recovery processes are identical and trans-diagnostic. The available evidence suggests that the effectiveness of peer support is not primarily dependent on shared diagnosis, but rather on transdiagnostic processes such as empowerment, hope, role modeling, and reduction of self-stigma (16–19). Strict diagnostic matching also presents practical limitations, as it could reduce the flexibility and accessibility of peer support services, given the diversity of patient needs and the limited number of trained peer support workers.

Regarding difficulties in emotional life and social withdrawal, our findings are consistent with the literature demonstrating a positive impact of peer support on reducing social isolation (42, 43). Data from Espairs Rhône from March 2020 to June 2022 show that isolation is one of the most frequently addressed themes during individual interviews with a peer support worker, alongside the origin and manifestation of disorders, spirituality, post-hospitalization, and medication (44). It appears that clinicians in psychosocial rehabilitation centers referred to peer support workers to improve patients’ quality of life in relation to their social relationships. Indeed, peer support workers can assist patients in connecting with the community and their social network, thereby combating self-stigmatization. Stigmatization, including self-stigmatization, is one of the factors contributing to patient isolation in psychiatry and contributes to decreasing their quality of life (45). The example of a peer support worker who has recovered from their disorder and the message of hope they convey are mechanisms for reducing this self-stigmatization (46).

Patients with low treatment adherence appear to be more easily referred to a peer support worker. It can be hypothesized that these referrals are made with the idea that peer support could improve this adherence. Indeed, most peer support workers are broaching the subject of psychopharmacological treatment during their follow-up (47). Furthermore, this referral is consistent with the literature, which finds an improvement in treatment adherence when supported by a peer support worker (48).

We noticed that patients referred to a peer support worker more often have their own personal housing. There are also fewer patients considered as vulnerable adults referred to peer support workers. These criteria suggest a population with less socioeconomic vulnerability. It is noteworthy that employment status, which is also an indicator of precariousness, does not appear as a significant factor in these analyses. It can be assumed that clinicians will not prioritize referring patients in more precarious situations to peer support workers, but rather to other forms of support, considered more urgent, such as social workers, housing programs, etc. This choice may reflect a clinical rationale, fundamental needs related to housing, financial security, or legal protection is viewed as a prerequisite before engaging in recovery-oriented interventions such as peer support. Yet, the effectiveness of peer support has also been demonstrated among populations experiencing socioeconomic vulnerability, such as individuals without stable housing (49). Peer support interventions in such context have demonstrated positive effects on social support and mental health outcomes. Programs, such as “Housing First”, have also shown that combining stable housing with peer support can foster recovery and reduce service disengagement among individuals with multiples vulnerabilities (50). The under-referral of patients in more precarious situations to peer support may reflect systemic and organizational practices, rather than a lack of relevance of the intervention for this population.

The remaining measured criteria have a small effect size, which limits their interpretation. For example, a stage of “preparation” on the STORI scale was found to be a predictive factor for No-referral to a peer support worker (-3% referral to peer support workers). This scale proposes five stages of recovery: moratorium, awareness, preparation, rebuilding, and growth. The “preparation” stage characterizes a patient who assesses their strengths and weaknesses and begins to establish a strategy to achieve their goals. Indications for peer support based on the stage of recovery appear relevant and align with the literature demonstrating better overall recovery, increased hope and empowerment among patients followed by a peer support worker (51, 52). Interpretation is also limited regarding the functioning scale, satisfaction with physical well-being, and ease in managing external relationships.

This work is also one of the first to provide a detailed description of the patient population followed-up by peer support workers in psychosocial rehabilitation centers. Our study population is comparable to those found in other studies using REHABase in terms of age, housing, educational level, and primary diagnoses (37, 53). However, it differs in terms of sex ratio, which is less masculine than in other studies using REHABase (54). It is noteworthy that peer support workers intervenes early in the care process, in more than half of cases as the first or second intervention (38.1% and 23.9% respectively). This may be one of the interventions considered more accessible and acceptable by patients at the beginning of their recovery process.

### 4.2 Study limitations

Our study comports limitations. This study only pertains to patients undergoing rehabilitation whose data were documented in REHABase, e.g. with a disproportionate sex ratio, and in centers employing a peer support worker at the time of data collection, which were only four among thirty centers (Lyon, Grenoble, Limoges and Poitiers). This may create two issues. First, our results may not be easily generalizable to populations with different sex ratio compositions and non-rehabilitation types of services, such as community mental health centers, crisis outpatient units (day hospitals, mobile teams), or inpatient units. Second, this raises the potential for selection bias where the current sample may not be representative of the target population.

Not all data is fully documented in the cohort for each participant, which limits the sample size. This may have led to a decrease in the study’s power, and some differences between the two groups may have gone unnoticed. In addition, despite our use of imputation techniques, the substantial amount of missing data also introduces the potential for selection bias.

In addition, the scales used in REHABase, although validated psychometrically, are not all validated in their French translation. This is the case for the ISMI, CGI, Birchwood’s Insight Scale (BIS), and the STORI. To assess self-stigmatization, the use of the Self-Stigma Scale-Short (SSS-S), validated in French (55), could be considered instead of the ISMI.

Finally, our analysis covers follow-ups conducted between 2016 and 2023, a period during which peer support was undergoing significant expansion in France. It reflects the orientation practices during this period and is not intended to establish orientation criteria. These practices will likely evolve over time with increasing knowledge about the impact of peer support follow-up. The study does not differentiate between the various types of follow-ups offered by peer support workers (assistance with advance directives in psychiatry, assistance with life story writing, formal or informal individual meetings, etc.). Additionally, it does not take into account some users who encounter peer support workers outside the rehabilitation center, for example through associations or other support programs, which are therefore not recorded in the database.

### 4.3 Implications for rehabilitation practices

It is possible to consider recommendations regarding the criteria to investigate when deciding on the referral of a patient to a peer support worker or not. The criteria highlighted in this study include living conditions, self-stigmatization, social withdrawal, diagnosis of NDD, mental well-being, low insight regarding the need for treatment, and the quality of social support perceived by the patient. Practically, our study can inform provisional referral criteria and triage tools by highlighting that the transdiagnostic processes, for instance self-stigmatization and low treatment adherence, may signal benefit from peer support. The observed socio-economic patterning of referrals underscores the need to audit equity of access and to ensure that peer support is not inadvertently reserved for less vulnerable groups, despite evidence of benefit in precarious populations (49, 50). At a policy level, these data support ongoing efforts to formalize national guidance on referral to a peer support workers.

Additional studies are needed to complete our findings. These results could be supplemented by examining other factors absent from our database such as extrinsic factors (clinician attitudes, service availability or peer support training). Furthermore, additional studies focusing on the type of support offered by peer support workers, and formalizing ways to refer based on this specific support, could be valuable. Similarly, it would be necessary to study these criteria in other primary care psychiatry services (inpatient units, mobile teams, community mental health centers, etc.). These results could get clinicians to question their referral practices and to (re)define criteria based on one hand, on the literature findings, and on the other hand, on their clinical judgment, in a field that remains largely unexplored. It also seems relevant to regularly implement a multidisciplinary reassessment to evaluate the efficiency of interventions and the feasibility of peer support follow-ups.

## 5 Conclusion

The growing number of patients benefitting from peer support accompanies an increase in research in this field. This work is an initial endeavor aimed at enhancing understanding regarding peer support interventions. These findings justify and encourage further investigation to explore the criteria guiding referral to peer support. Future research would benefit from adopting mixed-method designs, integrating qualitative approaches such as focus groups and interviews, in order to capture the perspectives of clinicians, peer support workers, and patients, and to complement the quantitative results reported here.

## Data Availability

The raw data supporting the conclusions of this article will be made available by the authors, without undue reservation.

## References

[1] Penney D. Defining “Peer Support”: Implications for Policy, Practice, and Research. (2018). Advocates for Human Potential Inc., Massachussetts Association for Mental Health. Available online at: https://www.mamh.org/library/defining-peer-support-implications-for-policy-practice-and-research (Accessed October 4, 2025).

[2] Shalaby RAH Agyapong VIO. Peer support in mental health: literature review. JMIR Ment Health. (2020) 7:e15572. doi: 10.2196/15572, PMID: 32357127 PMC7312261

[3] Corrigan PW. Understanding peerness in recovery-oriented mental health care. Psychiatr Serv. (2024) 75:597–9. doi: 10.1176/appi.ps.20230392, PMID: 38050446

[4] Anthony WA. Recovery from mental illness: The guiding vision of the mental health service system in the 1990s. Psychosocial Rehabil J. (1993) 16:1123. doi: 10.1037/h0095655

[5] Deegan PE. Recovery as a self-directed process of healing and transformation. Occup Ther Ment Health. (2002) 17:5–21. doi: 10.1300/J004v17n03_02

[6] Chamberlin J. The ex-patients’ movement: Where we’ve been and where we’re going. J Mind Behav. (1990) 11:323–36.

[7] Mental health action plan 2013–2020. Available online at: https://www.who.int/publications-detail-redirect/9789241506021 (Accessed February 7, 2024).

[8] Kaufman L Kuhn W Manser S. Peer Specialist Training and Certification Programs: A National Overview. Austin, TX: Texas Institute for Excellence in Mental Health, School of Social Work, University of Texas at Austin (2016). Available online at: http://sites.utexas.edu/mental-health-institute/ (Accessed March 20, 2024).

[9] De Gastines Clotilde. Les Expert.es du Vécu en pauvreté: 20 ans de regard critique et constructif. Education santé(2024). Available online at: https://educationsante.be/les-expert-es-du-vecu-en-pauvrete-20-ans-de-regard-critique-et-constructif/ (Accessed July 2, 2025).

[10] Watson E Repper J. Peer Support in mental health and social care services: Where are we now? Imroc (2024). Nottingham, UK. Available online at: https://www.imroc.org/publications/peer-support-in-mental-health-and-social-care-services-where-are-we-now (Accessed July 2, 2025).

[11] Demailly L. La professionnalisation de la pair-aidance en santé mentale en France:processus et enjeux. L'accompagnement par les pairs, sous la direction d'Eve Gardien, Presses Universitaires de Grenoble (2021) 75-91.

[12] Niard C Franck N. Apports de la pair-aidance aux dispositifs de santé mentale en France. Quelles formes de pair-aidance pour quels objectifs? Pratiques en Santé Mentale. (2020) 66e année:507. doi: 10.3917/psm.203.0050

[13] Poulsen CH Egmose CH Ebersbach B Hjorthøj C Eplov LF. The “Paths to everyday life” peer support intervention for adults with mental health difficulties versus service as usual in a Danish community setting - results from a randomized two-armed, multi-site, superiority trial. BMC Psychiatry. (2025) 25:695. doi: 10.1186/s12888-025-07011-y, PMID: 40646520 PMC12254959

[14] Weingarten R. The development of peer support in the Netherlands, Brazil, and Israel. Psychiatr Rehabil J. (2012) 35:476–7. doi: 10.1037/h0094584, PMID: 23276244

[15] Hegedüs A Burr C Pfluger V Sieg D Nienaber A Schulz M. Peer support worker training: Results of the evaluation of the Experienced Involvement training programme in Switzerland and Germany. Int J Ment Health Nurs. (2021) 30:451–60. doi: 10.1111/inm.12805, PMID: 33118298

[16] Lloyd-Evans B Mayo-Wilson E Harrison B Istead H Brown E Pilling S. A systematic review and meta-analysis of randomised controlled trials of peer support for people with severe mental illness. BMC Psychiatry. (2014) 14:39. doi: 10.1186/1471-244X-14-39, PMID: 24528545 PMC3933205

[17] White S Foster R Marks J Morshead R Goldsmith L Barlow S. The effectiveness of one-to-one peer support in mental health services: a systematic review and meta-analysis. BMC Psychiatry. (2020) 20:534. doi: 10.1186/s12888-020-02923-3, PMID: 33176729 PMC7657356

[18] Fuhr DC Salisbury TT De Silva MJ Atif N van Ginneken N Rahman A. Effectiveness of peer-delivered interventions for severe mental illness and depression on clinical and psychosocial outcomes: a systematic review and meta-analysis. Soc Psychiatry Psychiatr Epidemiol. (2014) 49:1691702. doi: 10.1007/s00127-014-0857-5, PMID: 24632847 PMC4167169

[19] Parker S Arnautovska U Korman N Harris M Dark F. Comparative effectiveness of integrated peer support and clinical staffing models for community-based residential mental health rehabilitation: A prospective observational study. Community Ment Health J. (2023) 59:459 70. doi: 10.1007/s10597-022-01023-8, PMID: 36057000 PMC9981709

[20] Johnson S Lamb D Marston L Osborn D Mason O Henderson C. Peer-supported self-management for people discharged from a mental health crisis team: a randomised controlled trial. Lancet. (2018) 392:40918. doi: 10.1016/S0140-6736(18)31470-3, PMID: 30102174 PMC6083437

[21] Badouin J Bechdolf A Bermpohl F Baumgardt J Weinmann S. Preventing, reducing, and attenuating restraint: A prospective controlled trial of the implementation of peer support in acute psychiatry. Front Psychiatry. (2023) 14:1089484. doi: 10.3389/fpsyt.2023.1089484, PMID: 36824670 PMC9941159

[22] Pfeiffer PN Heisler M Piette JD Rogers MAM Valenstein M. Efficacy of peer support interventions for depression: a meta-analysis. Gen Hosp Psychiatry. (2011) 33:2936. doi: 10.1016/j.genhosppsych.2010.10.002, PMID: 21353125 PMC3052992

[23] Collins R Firth L Shakespeare T. Very much evolving »: a qualitative study of the views of psychiatrists about peer support workers. J Ment Health. (2016) 25:27883. doi: 10.3109/09638237.2016.1167858, PMID: 27068009

[24] HAS. Développer le recours aux pairs-aidants(2023). Available online at: https://www.has-sante.fr/jcms/p_3488901/fr/grande-precarite-et-troubles-psychiques-fiche-outil-pairs-aidants (Accessed March 8, 2024).

[25] Boyer L Simeoni MC Loundou A D’Amato T Reine G Lançon C. The development of the S-QoL 18: A shortened quality of life questionnaire for patients with schizophrenia. Schizophr Res. (2010) 121:24150. doi: 10.1016/j.schres.2010.05.019, PMID: 20541912

[26] Boyer L Fernandes S Faugere M Richieri R Auquier P Fond G. The validity of the SQoL-18 in patients with bipolar and depressive disorders: A psychometric study from the PREMIUM project. J Clin Med. (2022) 11:743. doi: 10.3390/jcm11030743, PMID: 35160196 PMC8836740

[27] Boyer P. EGF. In: Guelfi JD, editor. L’évaluation clinique standardisée en psychiatrie, tome 1. France: Éditions Pierre Fabre (1996).

[28] Jones RM Gerritsen C Maheandiran M Simpson AIF. Validation of the clinical global impression-corrections scale (CGI-C) by equipercentile linking to the BPRS-E. Front Psychiatry. (2020) 11:180. doi: 10.3389/fpsyt.2020.00180, PMID: 32265753 PMC7100373

[29] Trousselard M Steiler D Dutheil F Claverie D Canini F Fenouillet F. Validation of the Warwick-Edinburgh Mental Well-Being Scale (WEMWBS) in French psychiatric and general populations. Psychiatry Res. (2016) 245:282-90. doi: 10.1016/j.psychres.2016.08.050, PMID: 27565700

[30] Misdrahi D Verdoux H Llorca PM Baylé FJ. Therapeutic adherence and schizophrenia: the interest of the validation of the French translation of Medication Adherence Rating Scale (MARS). L’Encephale. (2004) 30:409–10., PMID: 15597462

[31] Toupin J Cyr M Lesage A Valiquette C. Validation d’un questionnaire d’évaluation du fonctionnement social des personnes ayant des troubles mentaux chroniques. Rev Can Santé Mentale Communautaire. (1993) 12:143–56. doi: 10.7870/cjcmh-1993-0008

[32] Lecomte T Corbière M Laisné F. Investigating self-esteem in individuals with schizophrenia: Relevance of the Self-Esteem Rating Scale-Short Form. Psychiatry Res. (2006) 143:99108. doi: 10.1016/j.psychres.2005.08.019, PMID: 16725210

[33] Birchwood M Smith J Drury V Healy J Macmillan F Slade M. A self-report Insight Scale for psychosis: reliability, validity and sensitivity to change. Acta Psychiatr Scand. (1994) 89:627. doi: 10.1111/j.1600-0447.1994.tb01487.x, PMID: 7908156

[34] Ritsher JB Otilingam PG Grajales M. Internalized stigma of mental illness: psychometric properties of a new measure. Psychiatry Res. (2003) 121:3149. doi: 10.1016/j.psychres.2003.08.008, PMID: 14572622

[35] Andresen R Caputi P Oades LG. Stages of recovery instrument: development of a measure of recovery from serious mental illness. Aust New Z J Psychiatry. (2006) 40:972–80. doi: 10.1080/j.1440-1614.2006.01921.x, PMID: 17054565

[36] Ihaka R Gentleman R. R: A language for data analysis and graphics. J Comput Graphical Stat. (1996) 5:299314. doi: 10.1080/10618600.1996.10474713

[37] Barbalat G Plasse J Chéreau-Boudet I Gouache B Legros-Lafarge E Massoubre C. Contribution of socio-demographic and clinical characteristics to predict initial referrals to psychosocial interventions in patients with serious mental illness. Epidemiol Psychiatr Sci. (2024) 33:e2. doi: 10.1017/S2045796024000015, PMID: 38282331 PMC10894705

[38] Phillips RV van der Laan MJ Lee H Gruber S. Practical considerations for specifying a super learner. Int J Epidemiol. (2023) 52:1276 85. doi: 10.1093/ije/dyad023, PMID: 36905602

[39] Kia M. Jedha.co. Available online at: https://www.jedha.co/formation-ia/cross-validation:~:text=La%20cross%20validation%20ou%20validation,fonctionner%20sur%20de%20nouvelles%20donn%C3%A9es (Accessed March 20, 2024).

[40] Franck N Bon L Dekerle M Plasse J Massoubre C Pommier R. Satisfaction and needs in serious mental illness and autism spectrum disorder: the REHABase psychosocial rehabilitation project. Psychiatr Serv. (2019) 70:31623. doi: 10.1176/appi.ps.201800420, PMID: 30691384

[41] Burke C Hodgson C. Exploration of peer support models for individuals within Learning Disability and Neuro Diverse communities relating to mental health peer support. UK: Centre for Mental Health (2020).

[42] Castellanos D Capo M Valderrama D Jean-Francois M Luna A. Relationship of peer specialists to mental health outcomes in South Florida. Int J Ment Health Syst. (2018) 12:59. doi: 10.1186/s13033-018-0239-6, PMID: 30377441 PMC6195727

[43] Lien AD Meissen G. Peer perspectives: expectations and satisfaction with certified peer specialist services. Int J Psychosoc Rehabil. (2012) 17:5–16.

[44] Niard C Gross O Maugiron P Staedel B Franck N. Pair-aidance professionnelle. EMC Psychiatr. (2023) 39:1–13.

[45] Chiang YS Chang YC Liu YP Tzeng WC. Quality of life in patients with comorbid serious mental illness and chronic diseases: A structural equation model. J Adv Nurs. (2021) 77:127183. doi: 10.1111/jan.14663, PMID: 33230880

[46] Nguyen J Goldsmith L Rains LS Gillard S. Peer support in early intervention in psychosis: a qualitative research study. J Ment Health. (2022) 31:196202. doi: 10.1080/09638237.2021.1922647, PMID: 33961753

[47] Rodier P Franck N. Pair-aidance en santé mentale et adhésion médicamenteuse. Annales Médico Psychol Rev Psychiatr. (2023) 181:825 8. doi: 10.1016/j.amp.2023.07.002

[48] Proudfoot J Parker G Manicavasagar V Hadzi-Pavlovic D Whitton A Nicholas J. Effects of adjunctive peer support on perceptions of illness control and understanding in an online psychoeducation program for bipolar disorder: A randomised controlled trial. J Affect Disord. (2012) 142:98 105. doi: 10.1016/j.jad.2012.04.007, PMID: 22858215

[49] Miler JA Carver H Foster R Parkes T. Provision of peer support at the intersection of homelessness and problem substance use services: a systematic ‘state of the art’ review. BMC Public Health. (2020) 20:641. doi: 10.1186/s12889-020-8407-4, PMID: 32381086 PMC7203893

[50] Bean KF Shafer MS Glennon M. The impact of housing first and peer support on people who are medically vulnerable and homeless. Psychiatr Rehabil J. (2013) 36:48–50. doi: 10.1037/h0094748, PMID: 23477651

[51] Lyons N Cooper C Lloyd-Evans B. A systematic review and meta-analysis of group peer support interventions for people experiencing mental health conditions. BMC Psychiatry. (2021) 21:315. doi: 10.1186/s12888-021-03321-z, PMID: 34162340 PMC8220835

[52] Mak WWS Fu ACM Auyeung L Cheng WWL Chan RCH Tse SSK. Nine-month longitudinal impact of peer support workers’ Recovery attributes on service users’ Recovery in Hong Kong. Psychiatr Serv. (2021) 72:12827. doi: 10.1176/appi.ps.202000006, PMID: 34015963

[53] Lio G Ghazzai M Haesebaert F Dubreucq J Verdoux H Quiles C. Actionable predictive factors of homelessness in a psychiatric population: results from the REHABase cohort using a machine learning approach. Int J Environ Res Public Health. (2022) 19:12268. doi: 10.3390/ijerph191912268, PMID: 36231571 PMC9565981

[54] Dubreucq J Plasse J Gabayet F Faraldo M Blanc O Chereau I. Self-stigma in serious mental illness and autism spectrum disorder: Results from the REHABase national psychiatric rehabilitation cohort. Eur Psychiatry. (2019) 63:e13. doi: 10.1192/j.eurpsy.2019.12, PMID: 32093806 PMC7315867

[55] Golay P Martinez D Silva B Bonsack C. Validation psychométrique d’une échelle française d’auto- stigmatisation auprès d’un échantillon de patients souffrant de troubles mentaux: la Self-Stigma Scale-Short (SSS-S). Annales Médico-psychologiques, revue psychiatrique (2022) 180:899–904. doi: 10.1016/j.amp.2021.09.002

