# Supplementary material for: Predictive factors for referral to a peer support worker in psychosocial rehabilitation centers
Source: Front Psychiatry. 2025 Oct 13;16:1648718. doi: 10.3389/fpsyt.2025.1648718 (PMC12554742; doi:10.3389/fpsyt.2025.1648718)
Supplement: Supplementary file 1 [file Table1.docx]

**SUPPLEMENTARY TABLE Characteristics of patients included with (PS group) or without (nPS group) peer support referral, after imputation.**

^1^Mean pooled (sd pooled); n pooled (prop); ^2^OR = Odds Ratio, CI = Confidence Interval; ^3^RQTH = Reconnaissance de la qualité de travailleur handicapé; ^4^SQoL18 = Schizophrenia Quality of life; ^5^WEMWBS = Warwick-Edinburg mental well-being scale; ^6^EAS = Echelle d’autonomie sociale; ^7^SERS = Self-esteem rating scale; ^8^BIS = Birchwood insight scale; ^9^MARS = Medication adherence rating scale ; ^10^ISMI = Internalized stigma mental illness; ^11^STORI = Stage of recovery instrument

| **Variable** | **PS** *N = 134^1^* | **nPS** *N = 242^1^* | **OR** **(95% CI)***^2^* | **p-value** |
| --- | --- | --- | --- | --- |
| Age | 33.33 (9.93) | 32.64 (9.95) | 1.01 (0.99 to 1.03) | 0.522 |
| Sex (M = 1; F = 2; Others = 99), n (%) |  |  |  |  |
| *Male* | 77 (57.5%) | 140 (57.9%) | — |  |
| *Female* | 57 (42.5%) | 102 (42.1%) | 1.02 (0.66 to 1.56) | 0.942 |
| Level of education, n (%) |  |  |  |  |
| *Less than a bachelor's degree* | 33 (24.8%) | 81 (33.6%) | — |  |
| *Bachelor's degree or higher* | 101 (75.2%) | 161 (66.4%) | 1.54 (0.94 to 2.51) | 0.084 |
| Primary diagnosis (DSM-5), n (%) |  |  |  |  |
| *Neurodevelopmental disorder* | 31 (23.3%) | 23 (9.50%) | — |  |
| *Schizophrenia spectrum* | 62 (46.1%) | 106 (43.8%) | 0.43 (0.23 to 0.81) | **0.009** |
| *Bipolar disorder* | 24 (17.6%) | 33 (13.6%) | 0.53 (0.25 to 1.13) | 0.098 |
| *Other: anxiety disorder, depressive disorder, personality disorder* | 17 (13.0%) | 80 (33.1%) | 0.16 (0.08 to 0.34) | **<0.001*** |
| Secondary diagnosis, n (%) |  |  |  |  |
| *No* | 92 (68.4%) | 168 (69.4%) | — |  |
| *Yes* | 42 (31.6%) | 74 (30.6%) | 1.05 (0.66 to 1.67) | 0.835 |
| Number of psychoactive treatments, n (%) |  |  |  |  |
| *None* | 28 (20.7%) | 33 (13.6%) | — |  |
| *1 or 2* | 68 (50.6%) | 125 (51.7%) | 0.64 (0.36 to 1.17) | 0.147 |
| *3 or more* | 38 (28.7%) | 84 (34.7%) | 0.54 (0.29 to 1.03) | 0.061 |
| Global Assessment of Functioning, mean (SD) | 58.82 (14.28) | 57.94 (12.29) | 1.01 (0.99 to 1.02) | 0.550 |
| Clinical Global Impression (Severity scale), mean (SD) | 4.20 (1.20) | 4.14 (1.07) | 1.05 (0.85 to 1.31) | 0.649 |
| Marital status |  |  |  |  |
| *Single* | 102 (76.4%) | 185 (76.3%) | — |  |
| *In a relationship* | 32 (23.6%) | 57 (23.7%) | 0.99 (0.60 to 1.64) | 0.976 |
| Housing status, n (%) |  |  |  |  |
| *Homeless/Hospital/Squats* | 1.0 (0.75%) | 2.0 (0.83%) | — |  |
| *Personal home* | 81 (60.1%) | 143 (58.9%) | 1.13 (0.10 to 12.8) | 0.921 |
| *Family home* | 48 (36.0%) | 78 (32.3%) | 1.23 (0.11 to 14.1) | 0.866 |
| *Social care home/Others* | 4 (3.1%) | 19 (7.93%) | 0.44 (0.03 to 6.12) | 0.537 |
| Professional situation, n (%) |  |  |  |  |
| *Unemployed* | 116 (86.6%) | 214 (88.4%) | — |  |
| *Employed* | 18 (13.4%) | 28 (11.6%) | 1.18 (0.62 to 2.27) | 0.609 |
| Recognition of disabled worker status (RQTH^3^) |  |  |  |  |
| *No* | 78 (57.9%) | 136 (56.0%) | — |  |
| *Yes* | 50 (37.5%) | 101 (41.9%) | 0.87 (0.55 to 1.37) | 0.533 |
| *Pending request* | 6 (4.63%) | 5.0 (2.07%) | 2.16 (0.64 to 7.34) | 0.215 |
| Protection of Vulnerable Adults |  |  |  |  |
| *No* | 128 (95.4%) | 221 (91.5%) | — |  |
| *Yes (guardianship, curatorship, or reinforced curatorship)* | 6 (4.63%) | 21 (8.51%) | 0.52 (0.20 to 1.35) | 0.177 |
| Duration of the disease, year | 12.31 (9.96) | 11.87 (8.93) | 1.01 (0.98 to 1.03) | 0.667 |
| First contact with psychiatry, n (%) |  |  |  |  |
| *< 2 years ago* | 23 (17.0%) | 33 (13.6%) | — |  |
| *2-4 years ago* | 32 (23.6%) | 59 (24.5%) | 0.77 (0.36 to 1.65) | 0.495 |
| *5-9 years ago* | 18 (13.4%) | 44 (18.2%) | 0.59 (0.27 to 1.31) | 0.195 |
| *> 10 years ago* | 62 (46.0%) | 106 (43.6%) | 0.84 (0.45 to 1.60) | 0.603 |

| **Variable** | **PS** *N = 134^1^* | **nPS** *N = 242^1^* | **OR** **(95% CI)***^2^* | **p-value** |
| --- | --- | --- | --- | --- |
| Number of hospitalizations, n (%) |  |  |  |  |
| *None* | 30 (22.7%) | 47 (19.4%) | — |  |
| *1-3* | 69 (51.2%) | 138 (57.0%) | 0.77 (0.44 to 1.33) | 0.344 |
| *4-9* | 32 (23.9%) | 49 (20.3%) | 1.00 (0.50 to 2.00) | 0.990 |
| *10 or more* | 3 (2.24%) | 7.8 (3.22%) | 0.60 (0.14 to 2.45) | 0.472 |
| Duration of stay, n (%) |  |  |  |  |
| *None* | 30 (22.7%) | 47 (19.4%) | — |  |
| *< 6 months* | 70 (51.9%) | 130 (53.7%) | 0.83 (0.48 to 1.43) | 0.495 |
| *6 months- 1 year* | 16 (11.9%) | 36 (14.8%) | 0.68 (0.27 to 1.76) | 0.419 |
| ≥ *1 year* | 18 (13.4%) | 29 (12.1%) | 0.95 (0.44 to 2.08) | 0.902 |
| History of suicidal behaviors, n (%) |  |  |  |  |
| *No* | 101 (75.4%) | 160 (66.3%) | — |  |
| *Yes* | 33 (24.6%) | 82 (33.7%) | 0.64 (0.39 to 1.05) | 0.075 |
| Marginalization, n (%) |  |  |  |  |
| *No* | 127 (94.9%) | 222 (91.7%) | — |  |
| *Yes* | 7 (5.07%) | 20 (8.26%) | 0.59 (0.24 to 1.47) | 0.258 |
| Forensic history, n (%) |  |  |  |  |
| *No* | 127 (95.1%) | 223 (92.2%) | — |  |
| *Yes* | 7 (4.93%) | 19 (7.77%) | 0.61 (0.24 to 1.57) | 0.307 |
| Addiction: Tobacco, n (%) |  |  |  |  |
| *No* | 90 (66.9%) | 147 (60.9%) | — |  |
| *Yes* | 44 (33.1%) | 95 (39.1%) | 0.77 (0.49 to 1.22) | 0.263 |
| Addiction: Alcohol, n (%) |  |  |  |  |
| *No* | 108 (80.6%) | 202 (83.3%) | — |  |
| *Yes* | 26 (19.4%) | 40 (16.7%) | 1.20 (0.68 to 2.11) | 0.523 |
| Addiction: Psychoactive substance, n (%) |  |  |  |  |
| *No* | 118 (88.4%) | 196 (81.1%) | — |  |
| *Yes* | 16 (11.6%) | 46 (18.9%) | 0.56 (0.29 to 1.08) | 0.084 |
| SQoL18^4^ – Level of self-esteem (% of satisfaction) | 44.91 (29.98) | 43.42 (29.86) | 1.00 (0.99 to 1.01) | 0.650 |
| SQoL18^4^ – Level of resilience (% of satisfaction) | 55.11 (27.74) | 56.46 (26.62) | 1.00 (0.99 to 1.01) | 0.645 |
| SQoL18^4^ – Level of autonomy (% of satisfaction) | 59.91 (27.31) | 61.98 (25.80) | 1.00 (0.99 to 1.01) | 0.489 |
| SQoL18^4^ – Level of physical well-being (% of satisfaction) | 43.86 (27.35) | 39.74 (26.82) | 1.01 (1.00 to 1.01) | 0.179 |
| SQoL18^4^ – Quality of family relationships (% of satisfaction) | 64.70 (28.19) | 64.88 (27.64) | 1.00 (0.99 to 1.01) | 0.956 |
| SQoL18^4^ – Quality of relationships with friends (% of satisfaction) | 47.46 (28.96) | 54.32 (28.81) | 0.99 (0.98 to 1.00) | **0.032*** |
| SQoL18^4^ – Quality of sentimental life (% of satisfaction) | 34.29 (31.34) | 38.61 (32.22) | 1.00 (0.99 to 1.00) | 0.218 |
| SQoL18^4^ – Level of psychological well-being (% of satisfaction) | 47.36 (24.76) | 50.92 (24.39) | 0.99 (0.99 to 1.00) | 0.184 |
| SQoL18^4^ – Total score (% of satisfaction) | 49.71 (17.53) | 51.26 (17.82) | 1.00 (0.98 to 1.01) | 0.419 |
| WEMWBS^5^ – Total score (z-score) | -1.23 (1.25) | -1.36 (1.20) | 1.09 (0.91 to 1.29) | 0.354 |
| EAS^6^ - Personal Care Management | 3.91 (4.03) | 3.46 (3.62) | 1.03 (0.97 to 1.10) | 0.314 |
| EAS^6^ - Daily life management | 5.18 (5.23) | 4.92 (5.15) | 1.01 (0.96 to 1.06) | 0.692 |
| EAS^6^ - Resource management | 7.97 (5.05) | 7.64 (4.97) | 1.01 (0.97 to 1.06) | 0.571 |
| EAS^6^ - Management of external relations | 4.14 (3.90) | 4.22 (3.84) | 1.0 (0.93 to 1.06) | 0.868 |
| EAS^6^ - Management of emotional life and social relationships | 7.40 (3.74) | 6.45 (3.90) | 1.07 (1.01 to 1.13) | **0.024*** |
| EAS^6^ – Total score (score from 0 to 102) | 28.59 (14.81) | 26.68 (14.86) | 1.01 (0.99 to 1.02) | 0.257 |
| SERS^7^ – Positive self-esteem (score from 0 to 70) | 37.83 (11.40) | 38.21 (10.57) | 1.00 (0.98 to 1.02) | 0.752 |

| **Variable** | **PS** *N = 134^1^* | **nPS** *N = 242^1^* | **OR** **(95% CI)***^2^* | **p-value** |
| --- | --- | --- | --- | --- |
| SERS^7^ – Negative self-esteem (score from 0 to 70) | 38.98 (12.36) | 39.93 (11.57) | 0.99 (0.98 to 1.01) | 0.459 |
| SERS^7^ – Total score (score from -70 to 70) | -1.15 (19.86) | -1.72 (18.91) | 1.00 (0.99 to 1.01) | 0.787 |
| BIS^8^ – Attribution of symptoms (score from 0 to 4) | 2.75 (1.30) | 2.73 (1.27) | 1.02 (0.83 to 1.24) | 0.870 |
| BIS^8^ – Awareness of illness (score from 0 to 4) | 2.31 (1.33) | 2.34 (1.35) | 0.98 (0.80 to 1.21) | 0.868 |
| BIS^8^ – Need for treatment (score from 0 to 4) | 3.17 (0.98) | 3.36 (0.90) | 0.81 (0.64 to 1.03) | 0.084 |
| BIS^8^ – Total score (score from 0 to 12) | 8.24 (2.67) | 8.43 (2.48) | 0.97 (0.88 to 1.07) | 0.544 |
| MARS^9^ – Total score (score from 0 to 10) | 6.67 (2.14) | 6.65 (1.94) | 1.01 (0.90 to 1.12) | 0.926 |
| ISMI^10^ – Alienation (score from 1 to 4) | 1.84 (0.48) | 1.82 (0.44) | 1.12 (0.62 to 2.01) | 0.703 |
| ISMI^10^ – Perceived discrimination (score from 1 to 4) | 2.17 (0.67) | 2.13 (0.66) | 1.09 (0.76 to 1.57) | 0.631 |
| ISMI^10^ – Social withdrawal (score from 1 to 4) | 2.38 (0.65) | 2.28 (0.66) | 1.26 (0.85 to 1.86) | 0.243 |
| ISMI^10^ – Stereotype endorsement (score from 1 to 4) | 2.22 (0.51) | 2.18 (0.50) | 1.18 (0.74 to 1.90) | 0.485 |
| ISMI^10^ – Stigma resistance (score from 1 to 4) | 2.55 (0.58) | 2.56 (0.57) | 0.97 (0.67 to 1.41) | 0.876 |
| ISMI^10^ – Total score (score from 1 to 4) | 2.25 (0.46) | 2.22 (0.44) | 1.17 (0.69 to 1.99) | 0.561 |
| STORI^11^ – Moratorium (score from 0 to 50) | 20.34 (10.10) | 21.58 (9.78) | 0.99 (0.96 to 1.01) | 0.303 |
| STORI^11^ – Awareness (score from 0 to 50) | 24.05 (10.62) | 25.63 (9.73) | 0.98 (0.96 to 1.01) | 0.187 |
| STORI^11^ – Preparation (score from 0 to 50) | 24.61 (10.28) | 26.02 (10.00) | 0.99 (0.96 to 1.01) | 0.232 |
| STORI^11^ – Rebuilding (score from 0 to 50) | 28.67 (10.72) | 28.73 (10.09) | 1.00 (0.98 to 1.02) | 0.961 |
| STORI^11^ – Growth (score from 0 to 50) | 26.35 (12.46) | 25.88 (12.18) | 1.00 (0.98 to 1.02) | 0.744 |
| STORI^11^ – Recovery stage (highest stage), n (%) |  |  |  |  |
| *1 - MORATORIUM* | 26 (19.4%) | 58 (24.0%) | — |  |
| *2 - AWARENESS* | 21 (15.8%) | 31 (13.0%) | 1.51 (0.70 to 3.29) | 0.293 |
| *3 - PREPARATION* | 11 (8.51%) | 27 (11.1%) | 0.95 (0.37 to 2.42) | 0.916 |
| *4 - REBUILDING* | 38 (28.7%) | 70 (28.8%) | 1.24 (0.64 to 2.40) | 0.520 |
| *5 - GROWTH* | 37 (27.6%) | 56 (23.1%) | 1.49 (0.73 to 3.02) | 0.268 |
